# Effects of temperature and humidity on cerebrovascular disease hospitalization in a super-aging society

**DOI:** 10.1038/s41598-023-47998-6

**Published:** 2023-11-23

**Authors:** Shunichi Doi, Kihei Yoneyama, Toshiya Yoshida, Yasuhito Kawagoe, Michikazu Nakai, Yoko Sumita, Yuki Ishibashi, Masaki Izumo, Yasuhiro Tanabe, Tomoo Harada, Yoshihiro J. Akashi

**Affiliations:** 1 Division of Cardiology, Department of Internal Medicine, St. Marianna University School of Medicine, Kawasaki, Japan; 2 Department of Medical and Health Information Management, National Cerebral and Cardiovascular Center, Suita, Japan; 3 Clinical Research Support Center, University of Miyazaki Hospital, Miyazaki, Japan

**Keywords:** Environmental social sciences, Health care, Neurology, Risk factors

## Abstract

Weather conditions influence the incidence of cardiovascular disease. However, few studies have investigated the association between weather temperature and humidity and cerebrovascular disease hospitalizations in a super-aging society. We included 606,807 consecutive patients with cerebrovascular disease admitted to Japanese acute-care hospitals between 2015 and 2019. The primary outcome was the number of cerebrovascular disease hospitalizations per day. Multilevel mixed-effects linear regression models were used to estimate the association of mean temperature and humidity, 1 day before hospital admission, with cerebrovascular disease hospitalizations, after adjusting for air pollution, hospital, and patient demographics. Lower mean temperatures and humidity < 70% or humidity ≧ 70% are associated with an increased incidence of cerebrovascular disease hospitalization (coefficient, − 1.442 [− 1.473 to − 1.411] per °C, *p* < 0.001, coefficient, − 0.084 [− 0.112 to − 0.056] per%, *p* < 0.001, and coefficient, 0.136 [0.103 to 0.168] per %, *p* < 0.001, respectively). Lower mean temperatures and extremely lower or higher humidity are associated with an increased incidence of cerebrovascular disease hospitalization in a super-aging society.

## Introduction

Cerebrovascular disease is one of the leading causes of death and disability in the elderly worldwide^1^. Recently, the incidence of hospitalization due to cerebrovascular disease has increased among older patients^2^, with older age being recognized as a risk factor for cerebrovascular disease development. Japan’s transition into a super-aging has resulted in a growing number of its individuals developing cerebrovascular diseases^3^. Consequently, the prevention of cerebrovascular disease has taken on paramount importance in maintaining a high quality of life in older age.

The incidence of cerebrovascular disease is likely associated with ambient temperature, as suggested from a small number of studies from various countries^4,5^. Conversely, studies have also failed to establish temperature and humidity as significant risk factors of cerebrovascular disease. While weather is a recognized predictor of cardiovascular disease^6,7^, limited evidence exists regarding its relationship with cerebrovascular disease in Japan. Furthermore, it remains uncertain whether temperature, humidity, or related factors play a role in influencing cerebrovascular disease incidence, particularly in Japan. Moreover, existing guidelines provide only minimal recommendations regarding temperature and humidity as risk factors of cerebrovascular disease^8,9^.

Therefore, we conducted an observational study using a nationwide registry database to investigate whether the number of cardio-cerebrovascular disease hospitalizations was related to weather, temperature, and humidity in a super-aging society in Japan. The Japanese Registry of All Cardiac and Vascular Diseases (JROAD) database includes all patients with cardio cerebrovascular diseases who require hospitalization and constitutes a nationwide dataset in Japan. This study aimed to assess the relationship between temperature or humidity and the development of cerebrovascular disease to guide clinicians on weather recommendations and improve healthcare for an increasingly aging society.

## Results

Data were collected for 606,807 consecutive patients with cerebrovascular disease admitted to 715 acute care hospitals in Japan between 2015 and 2019. The patient characteristics and demographics are shown in Table 1.

**Table 1:** Baseline characteristics.

	All
Demographics (n = 606,807)	
Age, year median (IQR)	75.0 (66.0, 83.0)
Gender, male, n (%)	338,317 (55.8)
Cerebrovascular disease	
Ischemic stroke, n (%)	422,759 (69.6)
Cerebral hemorrhage, n (%)	140,966 (23.2)
Subarachnoid hemorrhage, n (%)	44,305 (7.3)
Coexisting condition	
Charlson Comorbidity Index, median (IQR)	2.0 (1.0, 3.0)
Weather	
Mean temperature 1 day before admission, °C, median (IQR)	17.2 (8.9, 23.0)
Mean humidity 1 day before admission, %, median (IQR)	69.0 (60.0, 79.0)

The number of cerebrovascular diseases overlapped in subgroups. IQR, interquartile range.

In this study population, the median age was 75.0 (66.0–83.0) years, and 55.8% of the participants were male. The incidence rates of ischemic stroke, cerebral hemorrhage, and subarachnoid hemorrhage were 69.6%, 23.2%, and 7.3%, respectively. The number of cerebrovascular diseases overlapped in subgroups. The median Charlson Comorbidity Index score was 2.0 (1.0, 3.0). The median mean temperature and humidity 1 day before hospital admission for stroke were 17.2 °C and 69%, respectively.

### Association between weather conditions and cerebrovascular disease hospitalizations

**Figure 1:**
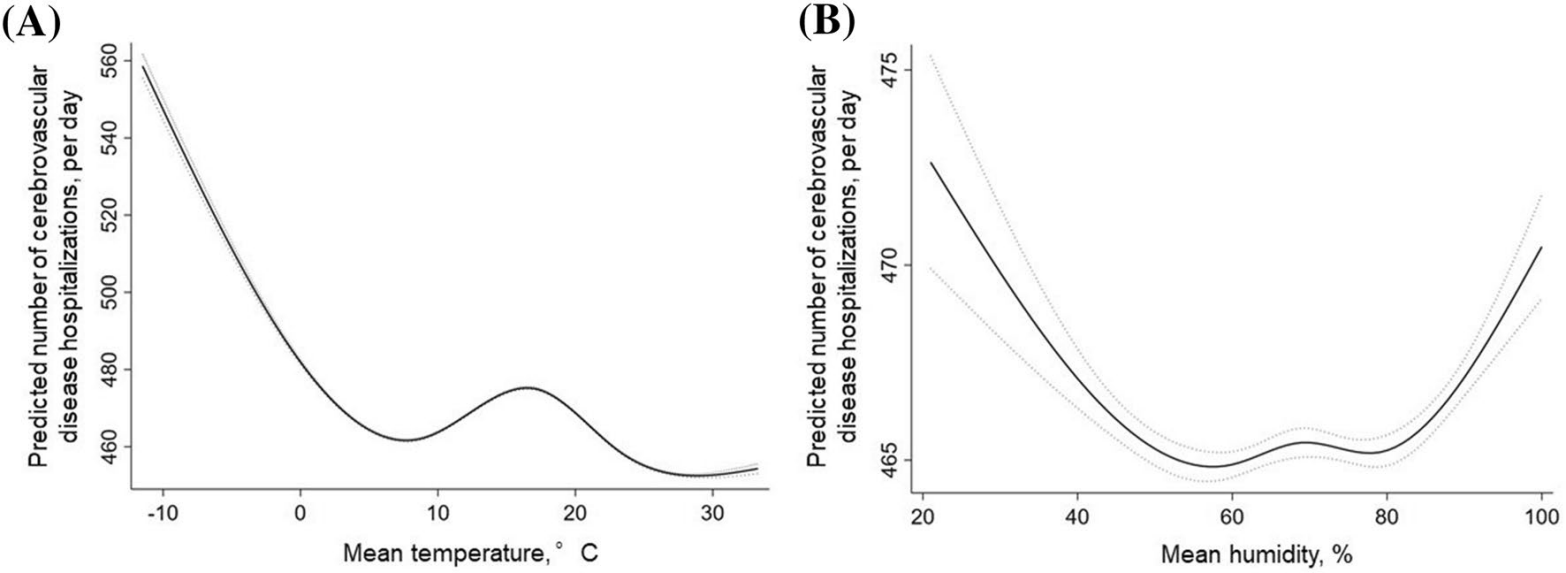
Association between mean temperature or humidity and cerebrovascular disease hospitalizations. (**A**) Linearity was checked for continuous and categorical variables using STATA’s multivariable regression splines (MVRS) command. MVRS indicated a non-linear relationship with mean temperature. The predicted number of cerebrovascular diseases per day was univariate. The x-axis represents temperature (°C) as a continuous variable. The solid and dashed lines indicate 95% confidence intervals. (**B**) MVRS indicated a non-linear relationship with humidity. The predicted number of cerebrovascular diseases per day was univariate. The x-axis represents humidity (%) as a continuous variable. The solid and dashed lines indicate 95% confidence intervals.

Multilevel mixed-effects linear regression analysis indicated that many incident cerebrovascular disease hospitalizations were associated with lower average temperature (coefficient, − 1.442 [− 1.473 to − 1.411] per °C (*P* < 0.001) after adjustments for season, PM2.5, hospital, and patient characteristics (age, sex, height, weight, smoking, and Charlson Comorbidity Index) (Table 2). The association between mean temperature and mean humidity for specific risk estimates of cerebrovascular disease incidence is summarized in Fig. 1. The number of cerebrovascular disease hospitalizations was higher with temperatures < 7 °C (coefficient, − 3.405 [− 3.547 to − 3.263]) (Fig. 1a).

**Table 2:** Association of the number of cerebrovascular disease hospitalization with temperature and humidity.

	Multilevel mixed-effects linear regression; random effect; institution	*p*-value
	Adjusted coefficient (95% confdence interval)	
All (cerebrovascular disease)		
Mean temperature (continuous values)	− 1.442 (− 1.473 to − 1.411)	< 0.001
Ischemic stroke		
Mean temperature < 7 °C	− 2.099 (− 2.221 to − 1.977)	< 0.001
Mean temperature ≧ 7 °C	0.191 (0.162 to 0.219)	< 0.001
Cerebral hemorrhage		
Mean temperature (continuous values)	− 0.091 (− 0.103 to − 0.079)	< 0.001
Subarachnoid hemorrhage		
Mean temperature (continuous values)	− 0.084 (− 0.112 to − 0.056)	< 0.001
All (cerebrovascular disease)		
Mean humidity < 70%	− 0.084 (− 0.112 to − 0.056)	< 0.001
Mean humidity ≧ 70%	0.136 (0.103 to 0.168)	< 0.001
Ischemic stroke		
Mean humidity < 40%	− 0.878 (− 1.107 to − 0.648)	< 0.001
Mean humidity ≧ 40%	0.165 (0.151 to 0.179)	< 0.001
Cerebral hemorrhage		
Mean humidity (continuous values)	− 0.142 (− 0.152 to − 0.133)	< 0.001
Subarachnoid hemorrhage		
Mean humidity (continuous values)	0.009 (0.003 to 0.015)	0.004

Coefficients represent the change in the number of cerebrovascular disease hospitalization per 1 unit increase in mean temperature or humidity, with adjustments for multiple variables: season, number of hospital beds, coronary care units, cardiac surgery, East/West Japan, age, sex, height, weight, Brinkmann Index, and Charlson Comorbidity Index score.

There was a non-linear relationship between humidity and the number of cerebrovascular disease hospitalizations (Fig. 1b). A negative linear association was found between mean humidity and cerebrovascular disease hospitalization at mean humidity < 70% (coefficient, − 0.084 [− 0.112 to − 0.056]). However, a positive linear association was found at mean humidity ≥ 70% (coefficient, 0.136 [0.103 to 0.168]).

### Effect of weather condition indices in the subgroups

**Figure 2:**
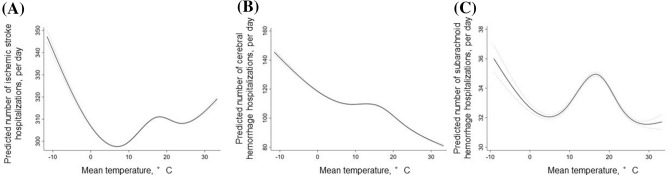
Association between mean temperature and subgroups of cerebrovascular disease hospitalizations. (**A**) Linearity was checked for continuous and categorical variables using STATA’s multivariable regression splines (MVRS) command. MVRS indicated a non-linear relationship with mean temperature. The predicted number of ischemic stroke hospitalizations per day was univariate. The x-axis represents temperature (°C) as a continuous variable. The solid and dashed lines indicate 95% confidence intervals. (**B**) MVRS indicated a non-linear relationship with temperature. The predicted number of cerebral hemorrhage hospitalizations per day was univariate. The x-axis represents temperature (°C) as a continuous variable. The solid and dashed lines indicate 95% confidence intervals. (**C**) MVRS indicated a non-linear relationship with temperature. The predicted number of cerebral hemorrhage hospitalizations per day was univariate. The x-axis represents temperature (°C) as a continuous variable. The solid and dashed lines indicate 95% confidence intervals.

There was a non-linear relationship between temperature and the number of ischemic stroke hospitalizations (Fig. 2a). A negative linear association was found between mean temperature and ischemic stroke hospitalization at mean temperature < 7 °C (coefficient, − 2.098 [− 2.220 to − 1.976]. However, a positive linear association was found at mean temperature ≥ 7 °C (coefficient, 0.190 [0.161 to 0.219].

**Figure 3:**
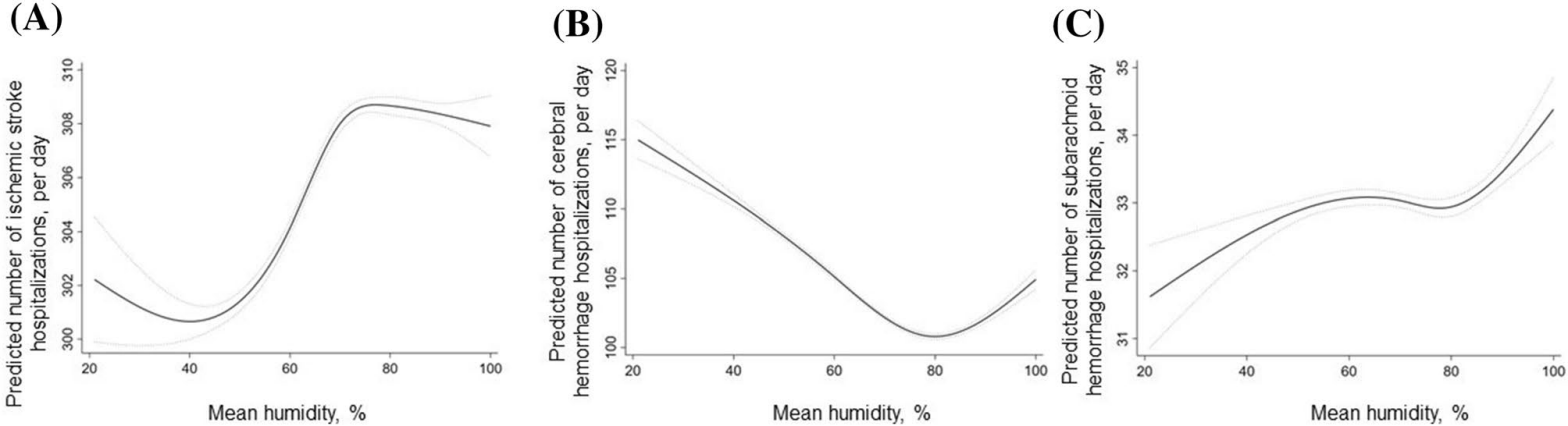
Association between mean humidity and subgroups of cerebrovascular disease hospitalizations. (**A**) Linearity was checked for continuous and categorical variables using STATA’s multivariable regression splines (MVRS) command. MVRS indicated a non-linear relationship with mean humidity. The predicted number of ischemic stroke hospitalizations per day was univariate. The x-axis represents humidity (%) as a continuous variable. The solid and dashed lines indicate 95% confidence intervals. (**B**) MVRS indicated a non-linear relationship with humidity. The predicted number of cerebral hemorrhage hospitalizations per day was univariate. The x-axis represents humidity (%) as a continuous variable. The solid and dashed lines indicate 95% confidence intervals. (**C**) MVRS indicated a non-linear relationship with humidity. The predicted number of cerebral hemorrhage hospitalizations per day was univariate. The x-axis represents humidity (%) as a continuous variable. The solid and dashed lines indicate 95% confidence intervals.

There was a non-linear relationship between humidity and the number of cerebrovascular disease hospitalizations (Fig. 3a). A negative linear association was found between mean humidity and ischemic stroke hospitalization at mean humidity < 40% (coefficient, 0.165 [0.151 to 0.179]. However, a positive linear association was found at mean humidity ≥ 40% (coefficient, 0.136 [0.103 to 0.168]).

The number of cerebral hemorrhage hospitalizations was higher with lower mean temperatures (coefficient, − 0.142 [− 0.151 to − 0.132] (Fig. 2b) and humidities (coefficient, − 0.142 [− 0.151 to − 0.132] (Fig. 3b). Also, the number of subarachnoid hemorrhage hospitalizations was higher with lower mean temperatures (coefficient, − 0.091 [− 0.103 to − 0.079] (Fig. 2c) and higher mean humidities (coefficient, 0.009 [0.003 to 0.015]) (Fig. 3c).

## Discussion

We present a contemporary analysis of nationwide data involving 606,807 patients and describe the estimated number of hospitalizations for incident cerebrovascular disease. Overall, a higher number of incident cerebrovascular hospitalizations was associated with (1) lower mean temperatures and (2) extremely lower or higher humidity. These findings highlight the importance of assessing lower mean temperatures and extreme humidity ranges as potential indicators for cerebrovascular disease risk, particularly in a super-aging society like Japan.

We found that lower mean temperatures were associated with increased hospitalization for cerebrovascular disease. Other studies have shown that cold-induced systemic hypertension^10^ is a risk factor associated with the renin-angiotensin system and sympathetic nerve activity, which could modulate the incidence of cerebrovascular disease hospitalization. Vascular compression of the rostral ventrolateral medulla (RVLM) is a known cause of hypertension^11,12^. Patients with RVLM vascular compression have shown greater variability in blood pressure during ischemic stroke^13^. Also, lower temperatures may be an environmental factor that causes a higher incidence of hospitalization.

Extremely lower or higher humidity was associated with increased hospitalizations due to cerebrovascular disease. However, a systematic review showed no association between daily humidity and cerebrovascular disease occurrence^14^. A previous study revealed that extremely lower or higher humidity correlated with the incidence of stroke^15^. High humidity can cause dehydration, which increases the risk of thrombosis. However, these influences may not be as strong as those of other weather conditions, such as temperature, which may be responsible for physiological changes that could increase stroke risk^16^. These include increased blood pressure, erythrocyte and thrombocyte counts, and blood viscosity during cold weather^17^. Plasma fibrinogen concentrations are also higher in older patients, especially during a cold weather^18^. This may partly explain why humidity is not associated with stroke occurrence.

These findings may be influenced by differences in the cerebrovascular disease type. Ischemic stroke has a higher incidence rate than cerebral or subarachnoid hemorrhage^19^. Our study might also show ischemic stroke as the main finding that revealed an association between a lower mean temperature, extremely lower or higher humidity, and hospitalization due to ischemic stroke. These results were consistent with previous reports^15^.

Elderly individuals tend to spend more time indoors due to factors such as illnesses and decreased physical function, compared to younger individuals. Therefore, there is a possibility that the impact of temperature differences may diminish with indoor activities. Nevertheless, limited research has compared indoor time between the elderly and younger age groups. Considering that the risk of illness is higher in the elderly, it is unclear whether temperature differences indoors have a significant effect on disease incidence. Although studies have reported differences in indoor and outdoor temperature and humidity^20^, these differences are generally consistent with seasonal trends. Specifically, in the context of Japan, it is important to note that regional variations and the presence or absence of heating and cooling systems can also influence these differences. However, there is currently no comprehensive report that has investigated these differences across all regions of Japan.

One of the limitations of our study is that we included only Japanese diagnosis procedure combination hospitals with cardiovascular beds that meet the JCS requirements. However, the JROAD is the largest retrospective study of nationwide cardiac health outcomes in Japan and constitutes a comprehensive database of epidemiological data for population-based studies. Two, retrospective studies do not determine cause and effect; however, the large sample size is a noteworthy strength of this study. Three, When patients are exposed to weather conditions different than that of the hospital they are admitted in, it could introduce bias in the results. Furthermore, this study does not include data on specific weather conditions observed at the research site, which could affect the outcomes. Finally, the predictor of cerebrovascular disease was not adjusted for hypertension.

## Conclusion

Lower mean temperatures and extremely lower or higher humidity are associated with an increased incidence of cerebrovascular disease hospitalization in the aging Japanese society. The findings of this study can provide guidance to healthcare providers on advising patients to monitor weather, temperature, and humidity in a country with an increasingly aging society.

## Methods

### Data collection

The JROAD database is a nationwide retrospective registry. The database was designed to assess the clinical activity of each Japanese institution regarding cardiovascular care and to provide adequate feedback to teaching hospitals for improving patient care. A detailed description of the database design and methods has been previously published^21^. The JCS developed the JROAD database, which includes the demographics of each hospital since 2004. The JCS also developed the JROAD-DPC nationwide database, which includes data from the Japanese diagnosis procedure combination/per diem payment system (DPC/PDPS) since 2014. The DPC database is a mixed-case classification system linked with a lump-sum payment system, launched in 2002 by the Japanese Ministry of Health, Labour and Welfare^22^. Compared with other registry databases, the Japanese DPC database enables researchers to conduct nationwide descriptive and/or analytical epidemiology studies in a real-world clinical setting. The JROAD database started to collect data about cerebrovascular disease in 2015. The DPC database includes data on the following elements: demographics for each patient (e.g., age and sex); principal diagnoses (coded according to the International Classification of Diseases, 10th revision [ICD-10]); comorbidities at admission (ICD-10 coded); complications after admission (ICD-10 coded); procedures, including surgery, medications, and devices used during hospitalization; length of stay; discharge status; and medical expenses^21–24^. Various institutions using the DPC system include academic, urban, and rural hospitals^21,24^. All data included in this study were from hospitalized patients with clinically apparent cardio-cerebrovascular disease. We collated and used a dataset of weather variables in Japan from the Japan Meteorological Agency (https://www.jma.go.jp/jma/indexe.html). The weather variables were daily weather temperatures and humidity, including maximal and minimum values. The humidity was measured as relative humidity. Localized weather data were obtained from the weather station closest to each hospital; as the hospitals were located in all 47 prefectures of Japan, weather data were obtained from all prefectural capitals. We searched the sites of weather stations by the municipality code, which the Ministry of Internal Affairs and Communications in Japan assigns to each municipality specifically. We combined the hospital sites with the monitoring stations in each prefecture to create a unified dataset. The weather variables were merged with the DPC database using the acute hospitalization day and the municipal code provided by the Japanese Ministry of Internal Affairs and Communications (https://www.soumu.go.jp/denshijiti/code.html). We also collated and used a dataset of air pollution variables in Japan from the National Institute for Environmental Studies (http://www.nies.go.jp/db/index-e.html). The air pollution variables included hourly PM2.5.

**Figure 4:**
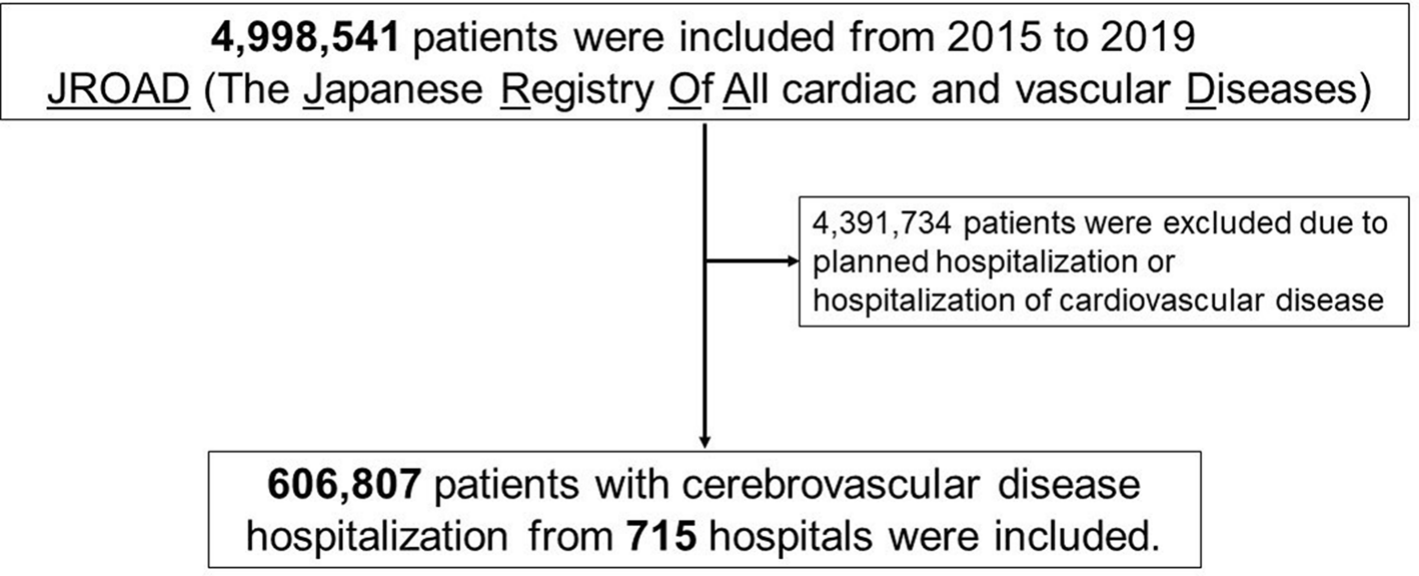
Flowchart of patient disposition. A total of 606,807 patients who underwent cerebrovascular disease hospitalization from 715 hospitals were included. The patients with planned hospitalization were excluded because only patients with acute hospitalized cerebrovascular disease were included for analysis. The patients with cardiovascular disease were also excluded.

This study included 715 hospitals and 4,998,541 consecutive patients admitted during the study period. We collected data from patients with cardio-cerebrovascular disease who required hospitalization. The exclusion criteria were as follows: cardiovascular disease hospitalization, planned hospitalization, missing temperature or humidity data, and missing patient characteristic data. After excluding 4,391,734 patients, a total of 606,807 patients with cerebrovascular disease were included in the analysis (Fig. 4).

The study was conducted in accordance with the principles of the Declaration of Helsinki. The author designed the present study, and the study protocol was approved by the Institutional Ethics Committee of St. Marianna University of Medicine. Each hospital anonymized the patient IDs using code-change equations for the original JROAD-DPC data. The requirement for individual informed consent was waived by the institutional ethics committee of St. Marianna University of Medicine because all data were anonymized when provided by the DPC. The data was sent to the Ministry of Health, Labor, and Welfare, Japan Meteorological Agency, and the National Cerebral Cardiovascular Center managed the database. The National Cerebral and Cardiovascular Center notified the patients that their information was being collected for this study through homepages or posters at each hospital. The patients could choose to exclude this information. This study did not investigate blood tests or electrocardiography data because we used only DPC data. The method of this study cited from previous our study^6,7^.

Cerebrovascular disease included ischemic stroke, cerebral hemorrhage, and subarachnoid hemorrhage. These diseases were assigned the International Statistical Classification of Diseases and Related Health Problems 10th Revision (ICD-10) code, which is recorded with the “main diagnosis, admission precipitating diagnosis, most resource-consuming diagnosis,” or “second-most resource-consuming diagnosis.” The number of cerebrovascular diseases overlapped in the subgroups. Ischemic stroke was assigned the ICD-10 code I63. Cerebral hemorrhage was assigned the ICD-10 code I61. Subarachnoid hemorrhage was assigned the ICD-10 code I60. The seasons were divided into spring (March–May), summer (June–August), autumn (September–November), and winter (December-February). The mean temporaries were defined as the mean hourly temperature and humidity within a day. The weather variables on a certain day were assigned to the day before the emergency hospitalization for cerebrovascular disease. This is because hospitalization times were not available in the database. The cutoff values associated with the results were visually determined by an author (S.D) from the graph.

### Design

We conducted a retrospective study using data from JROAD and JROAD-DPC and weather variables between April 1, 2015, and March 31, 2019.

### Outcomes

The primary outcome was the number of cerebrovascular diseases requiring hospitalization per day.

### Covariates

The mean weather temperature and humidity, as continuous variables, were adjusted for season, PM2.5, hospital demographics (East/West Japan, number of hospital beds, presence of a coronary care unit, cardiac surgery service, and board-certified cardiologist), and patient demographics (age, sex, height, weight, smoking, and Charlson Comorbidity Index). The Charlson Comorbidity Index has been developed and validated to predict the risk of mortality in longitudinal studies^25^. The score can be calculated from a weighted index consisting of age and the number and severity of comorbid diseases.

### Statistical analysis

Patient characteristics are expressed as medians and interquartile ranges for continuous variables. Categorical variables are presented as frequencies (%). We used multilevel mixed random-effects and population-averaged linear models to evaluate the association between the number of cerebrovascular disease hospitalizations and weather variables. Multilevel mixed-effects models were used to evaluate the random effects of hospital variations (institutional codes) using “xtset” command in STATA. Experienced cardiologists considered these covariates clinically important factors. As continuous variables, the weather temperature and humidity were adjusted for covariates.

Linearity was checked for continuous and categorical variables using STATA multivariate regression spline (MVRS) command. MVRS selects the regression spline model that best predicts the outcome variable. The MVRS indicated a linear spline relationship with humidity. The cutoff value of humidity was determined based on the knots calculated by MVRS and was tested using “mkspline” command in STATA. The mk-spline creates variables that contain a linear spline. These interactions were examined in each season. All analyses were performed using the STATA statistical software version 14 (Stata Corp., College Station, TX, USA). Statistical significance was defined as p < 0.05.

## Data Availability

The data that support the findings of this study are available from JROAD. However, restrictions apply to the availability of these data, which were used under the approval of the current study and are thus not publicly available. The data are available from JROAD upon reasonable request (jroad_dpcanaiysis@ml.ncvc.go.jp). Environmental data were obtained from the National Institute for Environmental Studies, Japan (http://www.nies.go.jp/db/index-e.html), and the Japan Meteorological Agency (https://www.data.jma.go.jp/obd/stats/etrn/index.php).
